# The impact of RNA binding motif protein 4-regulated splicing cascade on the progression and metabolism of colorectal cancer cells

**DOI:** 10.18632/oncotarget.5710

**Published:** 2015-10-19

**Authors:** Yu-Chih Liang, Wei-Cheng Lin, Ying-Ju Lin, Jung-Chun Lin

**Affiliations:** ^1^ School of Medical Laboratory Science and Biotechnology, College of Medical Science and Technology, Taipei Medical University, Taipei, Taiwan; ^2^ Division of Thoracic Surgery, Wan Fang Hospital, Taipei Medical University, Taipei, Taiwan; ^3^ School of Chinese Medicine, China Medical University, Taichung, Taiwan

**Keywords:** alternative splicing, colorectal cancer, miR-92a, nPTB, RBM4

## Abstract

Dysregulated splicing of pre-messenger (m)RNA is considered a molecular occasion of carcinogenesis. However, the underlying mechanism is complex and remains to be investigated. Herein, we report that the upregulated miR-92a reduced the RNA-binding motif 4 (RBM4) protein expression, leading to the imbalanced expression of the neuronal polypyrimidine tract-binding (nPTB) protein through alternative splicing-coupled nonsense mediated decay (NMD) mechanism. Increase in nPTB protein enhances the relative level of *fibroblast growth factor receptor 2 IIIc* (*FGFR2*) and *pyruvate kinase M2* (*PKM2*) transcripts which contribute to the progression and metabolic signature of CRC cells. Expression profiles of RBM4 and downstream alternative splicing events are consistently observed in cancerous tissues compared to adjacent normal tissues. These results constitute a mechanistic understanding of RBM4 on repressing the carcinogenesis of colorectal cells.

## INTRODUCTION

Alternative splicing (AS) functions as a prevalent mechanism in expanding the genetic diversity of eukaryotic cells [1]. Approximately 90% of human genes generate more than one transcript through this meticulously controlled process [2, 3]. The interplay between splicing factors and *cis*-elements constitutes the molecular mechanism in programming the splicing profile in spatiotemporal manners [4]. Altering expression levels or cellular localization directly changess the activity of splicing factors on AS events [5, 6]. The imbalanced expression or aberrant distribution of splicing factors is considered a common cause of hereditary diseases and cancers [7, 8]. An upregulated level of SRSF10 with a concomitant increase in the BCLAF1^+exon 5a^ isoform contributed to the active progression of colorectal cancer (CRC) [9]. An increase in polypyrimidine tract binding (PTB) protein induced the proliferation and progression of cancer cells compared with normal cell lines [10]. The imbalanced AS is a molecular occasion of carcinogenic signatures such as cellular immortality, invasion, and metastasis [11–13].

RBM4 was reported to reprogram a group of muscle-related genes and consequently facilitated differentiation of skeletal muscle cells [14]. During myogenesis, RBM4 synergized its influence by triggering the AS-coupled nonsense-mediated decay of the polypyrimidine tract-binding (PTB) protein and its paralog, neuronal (n)PTB [14]. In multiple cancer cells, RBM4 and PTB proteins exerted opposite effects on the carcinogenic signatures [15, 16]. RBM4 sensitized breast cancer cells toward a cytotoxic agent by modulating the splicing profile of the *insulin receptor* and *MCL-1* genes [17]. Overexpressing RBM4 antagonized the effect of oncogenic factors, including PTB and SRSF1 proteins in breast, lung, and prostate cancer cells [15].

PTB and nPTB are encoded by two paralogous genes and have highly similar RNA recognition motifs (RRMs), implying that they share RNA-binding properties [18]. Expression of PTB is ubiquitous but substantial among multiple tissues, whereas nPTB is mainly expressed in neuronal cells and testis [19, 20]. The extensively studied functions of the two PTB proteins are regulation of tissue-specific splicing events [21]. For instance, cross-regulation among miR-124, PTB, and nPTB constituted a molecular mechanism for the development of neuronal cells [22]. Upregulated expression of PTB and nPTB enhanced the progression of ovarian, breast cancer and glioma cells, and their expressions were highly relevant to the degree of malignancy [23–25]. The underlying mechanism for the cancer-associated upregulation of PTB/nPTB remained to be further investigated.

Herein, we assessed the influence of RBM4-regulated splicing cascade on the carcinogenic signature of CRC cells. Upregulated miR-92a reduced RBM4 expression by targeting to its coding region, which subsequently led to the increase in exon 10-included *nPTB* transcript in CRC tissues and cell lines. The RBM4-nPTB circuit modulated the invasion, migration, and mitochondrial activity of CRC cells by programming the splicing profiles of *FGFR2* and *PKM* genes.

## RESULTS

### RBM4 expression is reduced in cancerous tissues of CRC patients

**Figure 1:**
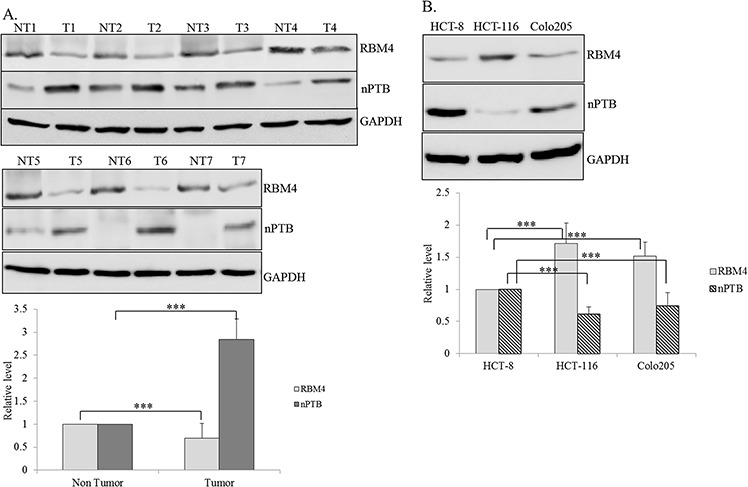
Differential expressions of RNA-binding motif 4 [RBM4] and neuronal polypyrimidine tract-binding protein [nPTB] in colorectal cancer [CRC] tissues and cell lines **A.** Protein extracts prepared from human CRC tissues [T] and adjacent non-cancerous tissues [NT] were subjected to an immunoblot assay using the indicated antibodies. The bar graph presents relative levels of RBM4 and nPTB in CRC tissues [*n* = 7]. **B.** Cell lysates isolated from distinct CRC cells were subjected to an immunoblot assay with the indicated antibodies. The bar graph shows relative levels of RBM4 and nPTB in three independent experiments using TotalLab Quant Software [*p < 0.05; **p < 0.01; ***p < 0.005].

RBM4 was demonstrated as a tumor suppressor in various malignancies, including breast, lung, ovarian, liver, and prostate cancer [15, 17]. To further investigate influences of RBM4 on distinct malignancies, such as CRC, its relevance in clinical tissue samples was first validated. Compared to adjacent normal tissues, reduced levels of the RBM4 protein were widely observed in cancerous tissues of CRC patients (Fig. 1A). A densitometric analysis of immunoblotting images showed about a 30% reduction in RBM4 in cancerous tissues (Fig. 1A, bar graph). Our previous study reported that RBM4 reduced the nPTB protein through the AS-coupled NMD in differentiating myocytes [14]. As expected, an increase in nPTB (~2.85-fold) was noted in cancerous tissues compared to adjacent normal tissues (Fig. 1A). The immunoblot results also revealed similar expression profiles of RBM4 and nPTB in distinct CRC cell lines, including HCT-8 and Colo205 cells as that of cancerous tissues (Fig. 1B). In contrast, the relatively high level of RBM4 with a concomitant decline in nPTB protein was observed in HCT-116 cells (Fig. 1B). The loss of RBM4 may result in the imbalanced expression of nPTB in CRC tissues and cells, which consequently reprogrammed splicing profiles in CRC cells.

### CRC-associated miRNA reduces the expression of RBM4

**Figure 2:**
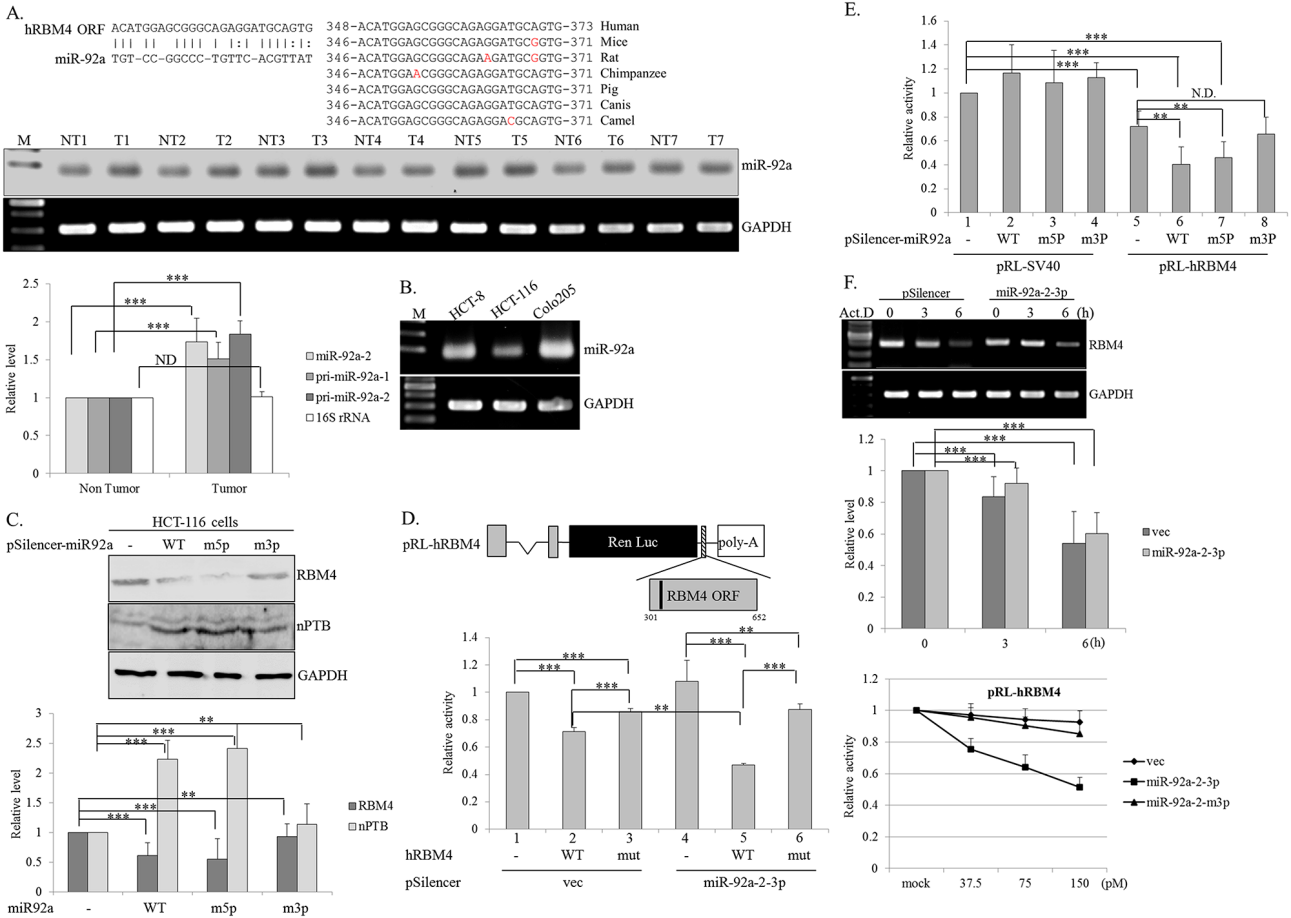
Targeting of miR92a-2-3p reduces the expression of RNA-binding motif 4 [RBM4] in colorectal cancer [CRC] cells **A.** The scheme represents the alignment between miR-92a and the non-canonical binding site within the human *RBM4* coding region [left]. The alignment result represents the conservation of the binding site within *RBM4* gene in different species [right]. Total RNAs extracted from [A] CRC tissues and **B.** CRC cell lines were polyadenylated using poly[A] polymerase. Expressions of miR92a in CRC tissues and cells were analyzed using an RT-PCR [*n* = 7]. The bar graph represents the expression profiles of miR92a, pri-miR-92-1/2, and 16S rRNA in 20 pairs of CRC tissues using a qRT-PCR analysis. **C.** The protein extract was prepared from cells transfected with the expression vector of WT miR-92a-2 and derived mutants, followed by an immunoblot assay using the indicated antibodies. The bar graph presents the relative level of nPTB and RBM4 using TotalLab Quant Software [*p < 0.05; **p < 0.01; ***p < 0.005]. **D.** The scheme shows the Renilla luciferase reporter containing the miR-92a targeting site [black line]. The intact pRL-h*RBM4* ORF reporter or its mutant was cotransfected with the expression vector of miR-92a-2 and the pFir-luc reference vector into HCT-116 cells. **E.** The intact pRL-h*RBM4* or pRL-SV40 reporter was cotransfected with the expression vector of WT miR-92a-2 and derived mutants into HCT-116 cells. The bar graph shows the relative Renilla luciferase activity normalized to firefly luciferase activity **F.** Abundances of *RBM4* transcripts in distinct groups were measured using an RT-PCR and qPCR assay. *In vitro* translation was performed with rabbit reticulocyte lysates and increasing amounts of overexpressing miR-92a-2 or the derived mutant. The translational activity was normalized to that in the reaction lacking overexpression of miR-92a. [*p < 0.05; **p < 0.01; ***p < 0.005].

RBM4 expression was controlled through multilayer regulation, including autoregulated AS and transcriptional control in differentiating brown adipocytes [27, 28]. However, microRNAs (miRNAs) constituted a posttranscriptional control in fine-tuning the protein expression profiles, which may also reduce RBM4 expression. The miRNA expression profiles in CRC tissues has been investigated using array analysis and further validated by quantitative approaches [29]. Forty-two upregulated CRC-related miRNAs (fold change > 2, *q* value < 0.05; 29) which contributed to CRC carcinogenesis were included for the following prediction (Supplementary Table 2). The folding energy of putative miRNA-*RBM4* pair was estimated using RNA22 V2.0 algorithm, a pattern-based program for identifying miRNA target site [30]. Supplementary Table 1 showed the predicted folding energies of thirteen miRNA-*hRBM4 pairs*, which were generated by aligning the sequence of *RBM4* coding region or 3′-UTR with the mature miRNAs sequence in RNA22 2.0 algorithm. The folding energies of these thirteen heteroduplexes were next calculated using RNAhybrid algorithm. One miR-92a targeting site was identified within the *RBM4* coding region (Fig. 2A, left) and two miR-17 targeting sites were predicted to reside in the coding region and 3′-UTR (Supplementary Table 1). The putative miR-92a binding site is conserved within *RBM4* transcripts in multiple species (Fig. 2A, right). In view of the low folding energy of miR-92a RBM4 pair (−19.2 Kcal/mol), the cross-species homology of miR-92a target site and the oncogenic effect of miR-92a on CRC [31], we next validated the effect of miR-92a on RBM4 reduction in CRC tissues and cells (Fig. 1A and 1B). A relatively high level of miR-92a was shown in cancerous tissues compared to adjacent normal tissues by a polyadenylation-coupled RT-PCR (Fig. 2A, *n = 7*). Results of real-time PCR analyses further indicated that the elevated miR-92a could be originated from primary miR-92a-1 and miR-92a-2 (*n* = 20, bar chart). The relatively high levels of miR-92a in HCT-8 and Colo205 cells (Fig. 2B) were relevant to the reduced RBM4 levels in those cells (Fig. 1B) even though the influence of miR-92a on RBM4 expression may have varied in the colorectal adenocarcinoma-derived (HCT8) and carcinoma-derived (Colo205) cell lines. To evaluate the targeting specificity of miR-92a toward *RBM4* transcripts, the miR-92a-2 expression vector was mutated by guanine-to-thymidine or adenine-to-cytosine substitutions at the second nucleotide within the seed sequence of miR-92a-2-5p and miR-92a-2-3p. Immunoblotting results showed the reduction of endogenous RBM4 protein with a concomitant increase in the nPTB protein in the presence of overexpressing miR-92a-2-3p (Fig. 2C, WT and m5p), but not miR-92a-2-5p (Fig. 2C, m3p) in HCT-116 cells. We next explored the validity of the bioinformatic forecast using *in vivo* and *in vitro* translation assays in turn. The Renilla luciferase reporter containing the miR-92a targeting site within the coding region of the *hRBM4* gene was constructed, and the derived mutant was next made by the thymidine -to-guanosine substitution in the second nucleotide of this targeting site. The activities of WT or mutant reporter were examined in the presence of empty or miR-92a-2-3p expressing vector in HCT-116 cells which expressed a relatively low level of miR-92a. The presence of the miR-92a targeting site reduced the activity of Renilla luciferase to about 71.4% of the level of the original reporter (Fig. 2D, lanes 1 and 2), whereas the single-nucleotide substitution of the miR-92a targeting element (Fig. 2D upper, T372G) partially restored the reduced activity (lane 3). Overexpressing miR-92a-2-3p further reduced the activity of the Renilla reporter containing the targeting site (lane 5), but not the derived mutant (lane 6). Moreover, the single-nucleotide substitution of miR-92a-2-3p, but not miR-92a-2-5p, relieved its repressive effect on the activity of the Renilla reporter containing the miR-92a target site (Fig. 2E, lane 8).

Imperfect base-pairing between microRNA and the non-canonical binding site was widely identified to preferentially stall translation, instead of inducing degradation of the target gene [32]. To verify the molecular mechanism regarding the miR-92a-caused reduction of RBM4, the stability of *RBM4* transcripts was validated in empty vector- or miR92a-2-3p-overexpressing cells after treatment with actinomycin D. Using RT-PCR and qPCR analyses, overexpression of miR-92a-2-3p had no distinct effect on the stability of *RBM4* transcripts (Fig. 2F, upper and middle). In contrast, the cytoplasmic extract containing the overexpressing WT miR92a-2-3p (Fig. 2F, lower, square), but not the derived mutant (triangle), substantially reduced the translation of *in vitro-*transcribed RNA containing the miR-92a binding site. These results demonstrated the mechanistic role of miR-92a on RBM4 reduction in CRC tissues and cells.

### RBM4 enhances exclusion of the nPTB exon 10 in CRC cells

**Figure 3:**
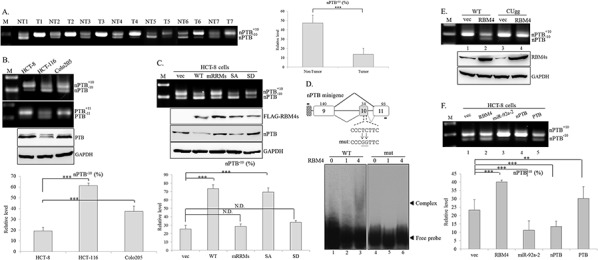
RNA-binding motif 4 [RBM4] enhances the skipping of *neuronal polypyrimidine tract-binding protein* [*nPTB*] exon 10 in colorectal cancer [CRC] cells Total RNAs extracted from **A.** paired CRC tissues and **B.** CRC cell lines were analyzed by an RT-PCR with a specific primer set against the *nPTB* and *PTB* genes. Western blotting was performed with the indicated antibodies. **C.** Total RNAs were extracted from HCT-8 cells transfected with FLAG-tagged RBM4 and derived mutants. The splicing profile of *nPTB* and its protein expression were analyzed as previously described. The bar graph presents the relative level of *nPTB^−10^* transcripts using TotalLab Quant Software [*p < 0.05; **p < 0.01; ***p < 0.005]. **D.** The diagram presents the sequence of the CU-element within *nPTB* exon 10. The mutant reporter contained pyrimidine-to-guanine nucleotide substitutions in the CU element. The mock eluate or recombinant His-tagged RBM4 protein was incubated with DIG-labeled probes. Mixtures were fractionated in 8% native acrylamide gels and transferred to Nylon membranes, followed by probing with an HRP-conjugated anti-DIG Fab fragment. **E.** The WT *nPTB* minigene or CUgg mutant was cotransfected with the empty vector or FLAG-RBM4 expression vector into HCT-8 cells. The PCR product of the spliced transcript was analyzed using electrophoresis on 2% agarose gels. **F.** The *nPTB* minigene was cotransfected with various expressing vectors into HCT-8 cells. The spliced transcript of the *nPTB* minigene was analyzed as described in the previous panel. The bar graph shows the relative level of *nPTB^−10^* in three independent experiments using TotalLab Quant Software [*p < 0.05; **p < 0.01; ***p < 0.005].

AS-coupled NMD pathway constituted a molecular mechanism leading to the tissue-restricted expression of the nPTB protein [33]. As shown in Fig 3A, a substantial decrease in the *nPTB^−10^* transcript, the AS-NMD substrate, was widely observed in CRC tissues compared to adjacent normal tissues (Fig. 3A, left). Densitometric analysis of RT-PCR images showed a 71% reduction in the *nPTB^−10^* transcript (non-tumor: 47%; tumor: 14%) in cancerous tissues (Fig. 3A, right). Fig. 3B shows different splicing patterns of *nPTB* and its paralog, *PTB*, in CRC cell lines. The majority of *nPTB* and *PTB* transcripts were *nPTB^+10^* and *PTB^+11^* in HCT-8 and Colo205 cells, whereas relatively high levels of the *nPTB^−10^* and *PTB^−11^* transcripts were noted in HCT-116 cells. Protein levels of nPTB and PTB were relevant to its splicing profile in the CRC cell lines (Fig. 1B and 3B). The expression profile of the *nPTB* gene may be correlated with the RBM4 abundance in the CRC cell lines. To verify this inference, the splicing pattern of *nPTB* was validated in the presence of FLAG-tagged RBM4 and the derived mutants. RT-PCR results and the densitometric analysis showed that the overexpression of WT RBM4 and nonphosphorylatable S309A protein (SA), but not phosphormimetic S309D (SD) and the RRM mutant containing four mutations, enhanced skipping of *nPTB* exon 10 in HCT-8 cells (Fig. 3C, upper and lower). Influences of overexpressing RBM4 proteins on the splicing pattern of *nPTB* pre-mRNA and the protein level were dependent on its biological activity instead of expression level (Fig. 3C, middle). The cytoplasm-enriched SD mutant was incompetent at mRNA processing, whereas the nuclear-localized SA mutant functioned normally as ordinary RBM4 [14, 17].

Our previous studies documented that the effect of RBM4 on the regulated exon changed with localization of the RBM4-responsive element [14, 17, 27, 28]. RBM4 enhanced the inclusion of alternatively spliced exons by directly binding to intronic elements close to it [34], but the simultaneous binding of RBM4 to the cassette exon and its flanking intron via the CU-element resulted in its skipping [14, 17, 27]. To demonstrate this speculation, the *nPTB* minigene and a derived mutant containing the cytosine and thymidine-to-guanine nucleotide substitutions in exon 10 were constructed (Fig. 3D). The binding between recombinant RBM4 and the exonic CU-element originating from *nPTB* exon 10 and intron 10 (120 nt) was examined using REMSA. The WT probe and RBM4 protein formed ribonucleoprotein complexes in a dose-dependent manner (Fig. 3D, lanes 2 and 3), whereas the guanine nucleotide substitutions within the CU-rich element reduced complex formation (Fig. 3D, mut). Overexpression of RBM4 induced the skipping of *nPTB* exon 10 within the WT minigene (Fig. 3E, lane 2), but had almost no effect on the mutant *nPTB* exon 10 (Fig. 3E, lane 4). These results indicated that a direct binding between RBM4 and the exonic CU-element reduced the utilization of *nPTB* exon 10. Overexpression of PTB protein exerted similar activity to RBM4 of repressing *nPTB^+10^* transcripts which originated from the *nPTB* minigene (Fig. 3F, lanes 2 and 5) as previously reported [22], and overexpressing miR-92a-2 inversely enhanced the production of *nPTB^+10^* transcripts (lane 3). Overexpressing nPTB constitute a feed-forward circuit which enhanced its exon 10-included transcripts in the CRC cells (lane 4).

### RBM4 exerts an opposite effect to nPTB on splicing profiles of CRC-related genes

**Figure 4:**
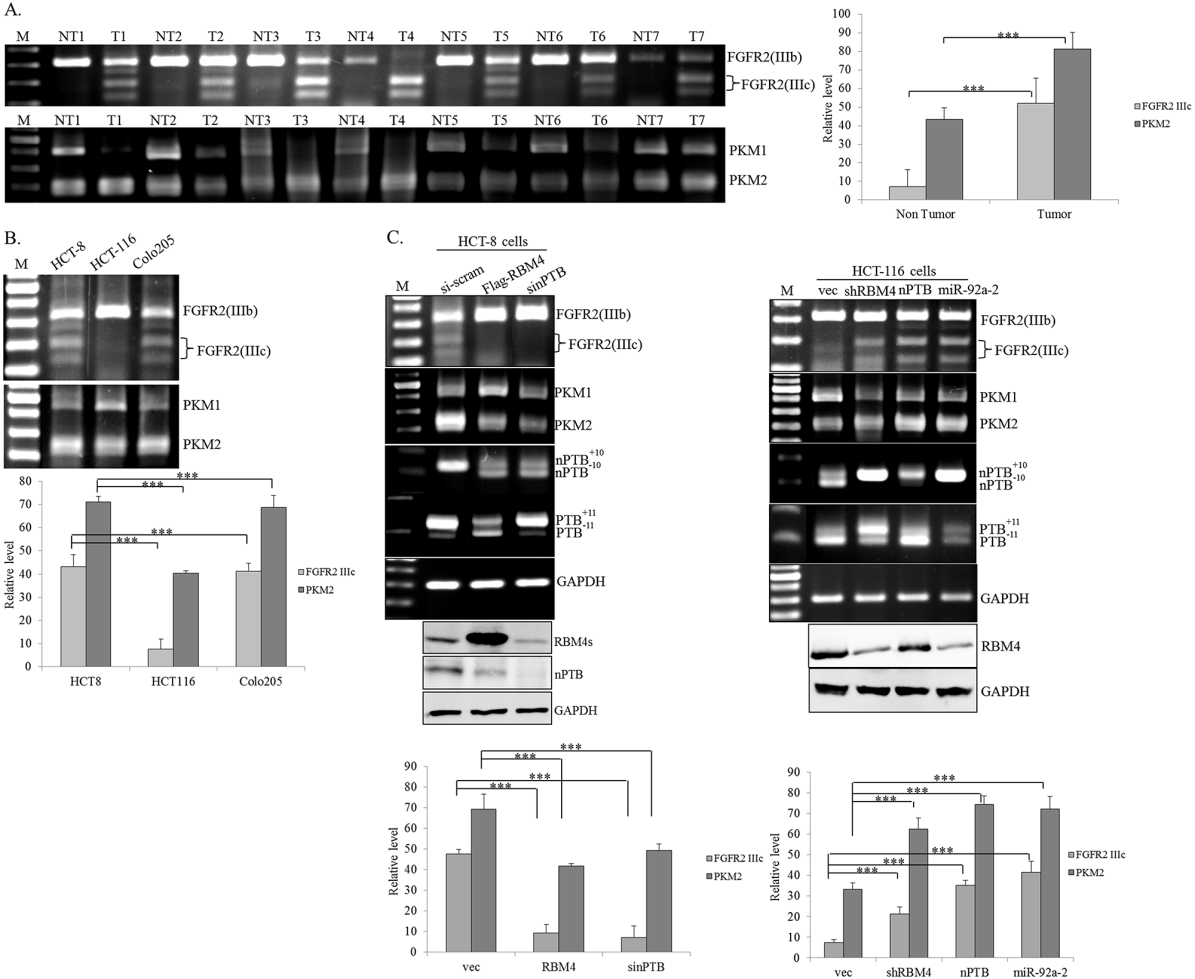
RNA-binding motif 4 [RBM4] and neuronal polypyrimidine tract-binding protein [nPTB] exerted opposite effects on the splicing patterns of the *FGFR2* and *PKM* genes Total RNA extracted from **A.** paired colorectal cancer [CRC] tissues and **B.** CRC cell lines were analyzed using an RT-PCR with specific primer sets. **C.** HCT-8 and HCT-116 cells were transfected with overexpressing or targeting vectors or synthesized siRNA, followed by total RNA extraction. Splicing profiles of *FGFR2* and *PKM* pre-mRNAs were analyzed as described in Materials and methods section. The bar graph shows relative levels of *FGFR2 IIIc* and *PKM2* in three independent experiments using TotalLab Quant Software [*p < 0.05; **p < 0.01; ***p < 0.005].

nPTB was demonstrated as a CRC-related oncogene in recent reports [35], but its effect on the CRC-associated splicing network is largely unknown. Around 74% similarity between the RRMs sequence of nPTB and PTB implies that nPTB possibly exerts a similar specificity to PTB-regulated splicing events, such as *FGFR2* and *PKM* in CRC cells [36–38]. The RT-PCR assay showed relatively high levels of *FGFR2 IIIc* and *PKM2* in cancerous tissues of CRC patients compared to adjacent normal tissues (Fig. 4A). The relatively high levels of *FGFR2 IIIc* and *PKM2* transcripts were noted in HCT-8 and Colo205 cells, but not in HCT-116 cells (Fig. 4B), which may be correlated to the expression profiles of RBM4 and nPTB proteins (Fig. 1B). Fig. 4C shows that RBM4 overexpression and nPTB knockdown distinctly reprogrammed the CRC-related splicing profiles including *FGFR2*, *PKM*, *PTB*, and *nPTB* transcripts in HCT-8 cells (left panel). In contrast, overexpression of nPTB, miR-92a-2, PTB or RBM4 knockdown induced the production of *FGFR2 IIIc*, *PKM2*, *nPTB^+10^*, and *PTB^+11^* transcripts which were ordinarily recessive in HCT-116 cells (right panel; Supplementary Fig. 1). Results of Fig. 3 and 4 suggested that the complex interplay among RBM4, PTB, and nPTB reprogrammed the CRC-associated AS cascade.

### Isoform switching of FGFR2 alters activities of downstream signaling proteins

**Figure 5:**
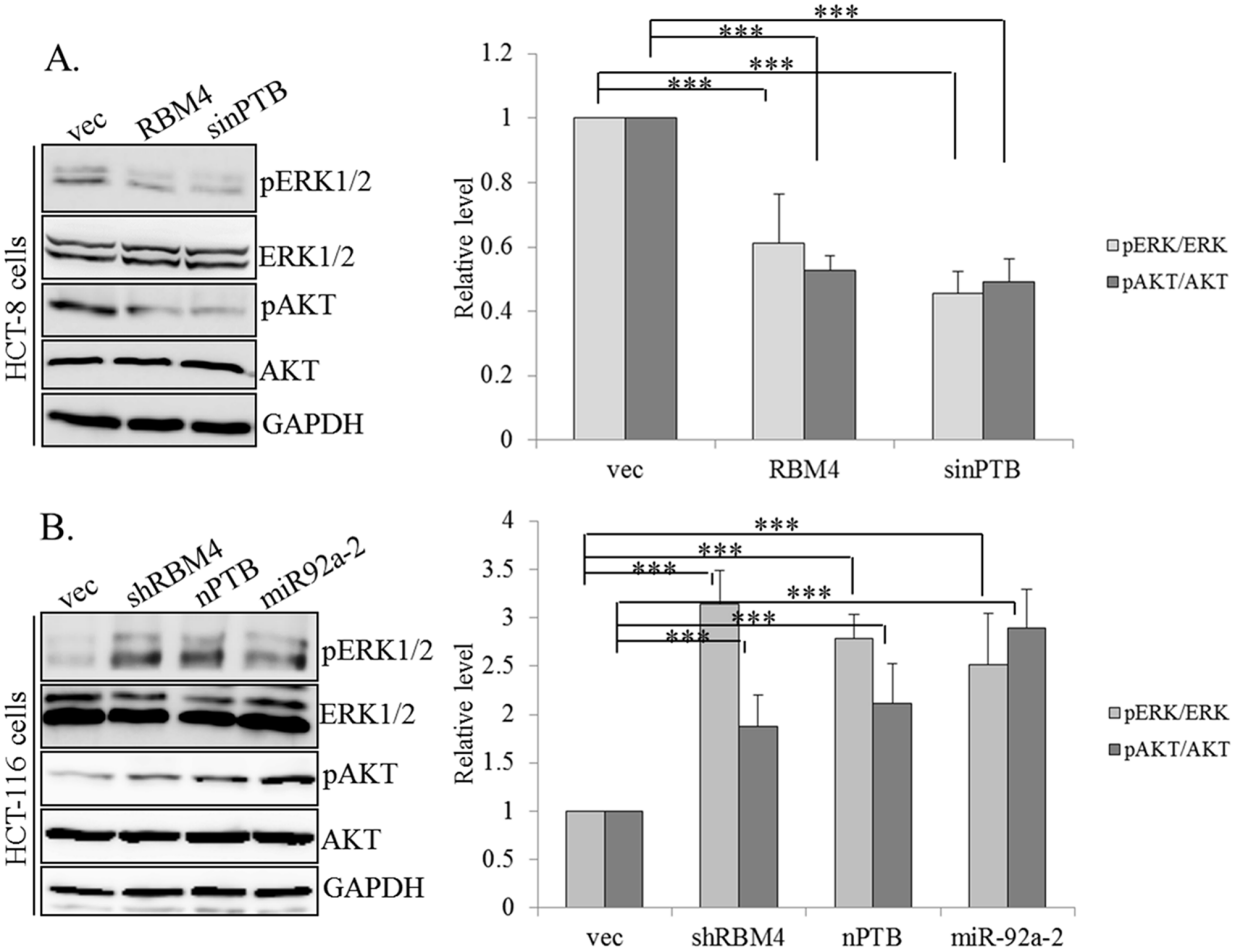
RNA-binding motif 4 [RBM4]-regulated splicing cascade modulates the activity of AKT/ERK signaling **A.** HCT-8 cells were transfected with an RBM4-expressing vector or siRNA against neuronal polypyrimidine tract-binding protein [nPTB]. **B.** HCT-116 cells were transfected with an nPTB- or miR92a-2-expressing vector or targeting vector of RBM4. The protein extract was extracted followed by an immunoblot analysis with the indicated antibodies. The bar graph presents the relative levels of phosphor-AKT and ERK protein using TotalLab Quant Software in three independent experiments.

Dysregulated signaling pathways, including active extracellular signal-regulated kinase (ERK) and AKT (or protein kinase B) greatly enhanced progression of CRC cells [39–42]. Upregulation of FGFR2 was reported to participate in activation of these two molecules in invasive cancer cells [43]. We wondered whether the RBM4-regulated splicing cascade changed the activities of these signaling pathways. Expression profiles of ERK and AKT were examined using specific antibodies which recognized the total or phosphorylated ERK and AKT proteins. In HCT-8 cells, RBM4 overexpression exerted a similar effect to that of nPTB knockdown on reducing phosphorylation of ERK/AKT without altering total levels of these two proteins (Fig. 5A, left). Quantitative results showed that the phosphorylated ERK/AKT in RBM4-overexpressing or nPTB-KD HCT-8 cells was downregulated to 50%~60% of empty vector-transfected cells (Fig. 5A, right). Conversely, overexpression of nPTB or miR-92a-2 or RBM4 knockdown induced phosphorylation of ERK/AKT in HCT-116 cells (Fig. 5B, left). Quantitative results of immunoblot images indicated that phosphorylated ERK/AKT was induced to about 2~3-fold of empty vector-transfected cells (Fig. 5B, right). These results suggested that RBM4 and nPTB exerted antagonistic effects on programming the splicing profile of FGFR2 and subsequently changed the activity of its downstream signaling pathway.

### RBM4 and nPTB differentially regulate invasive and migratory activities of CRC cells

**Figure 6:**
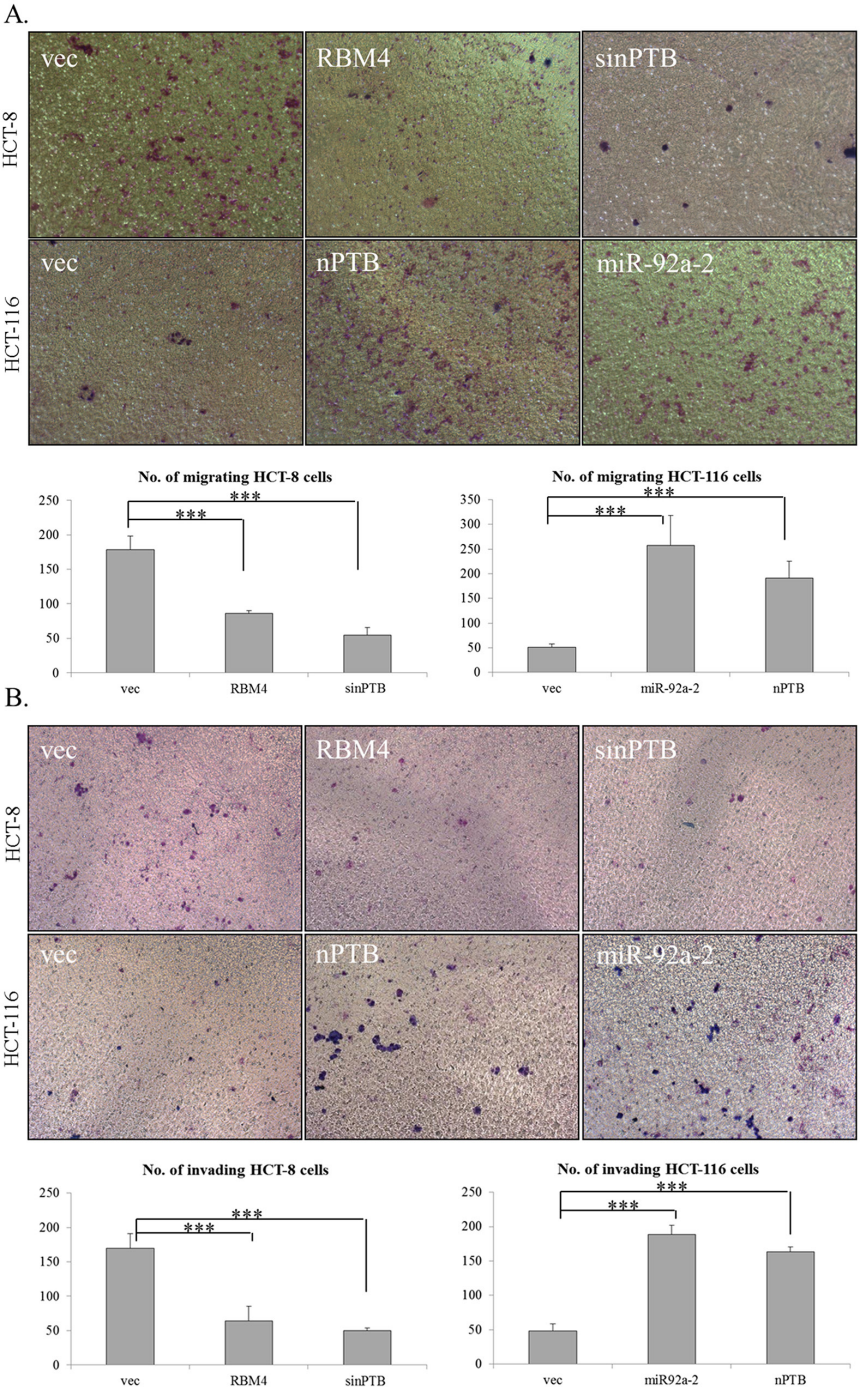
Expression profiles of RNA-binding motif 4 [RBM4] and neuronal polypyrimidine tract-binding protein [nPTB] influence the progression of colorectal cancer [CRC] cells **A.** The motility of HCT-8 and HCT-116 cells transfected with distinct expressing vectors or synthesized siRNA was analyzed using a transwell system, followed by Giemsa staining. **B.** HCT-8 and HCT-116 cells were transfected with a parallel set of expressing vectors or siRNA and seeded onto Matrigel at 24 h post-transfection. Penetrating cells were fixed and stained with Giemsa solution. Images were taken of penetrated cells on the filters. The counting results of three independent experiments are presented in a bar chart as the mean ± STD.

AKT/ERK signaling was reported to constitute a molecular mechanism in controlling the progression of CRC cells [39–42]. The motility of CRC cells may be correlated with the expression profiles of RBM4 and nPTB due to their influence on the phosphorylation of AKT/ERK proteins. To investigate this hypothesis, a migration/invasion assay was performed with distinct CRC cells transfected with distinct expressing or targeting vectors. Compared to empty vector-transfected HCT-8 cells, the staining images of transwell assay showed that the migratory activity of HCT-8 cells was reduced in the presence of overexpressing RBM4 or with ablation of endogenous nPTB (Fig. 6A, upper). The number of RBM4-overexpressing or nPTB-knockdown HCT-8 cells which migrated through the membrane was respectively reduced to ~50% or 30% of empty vector-transfected cells (Fig. 6A, lower). On the contrary, overexpression of nPTB or miR-92a-2 enhanced the migratory activity of HCT-116 cells compared to empty vector-transfected cells (Fig. 6A, upper). The number of migrating HCT-116 cells was elevated to ~5.7- or 3.6-fold of empty vector-transfected cells (Fig. 6A, lower). Similarly, staining images of the transwell assay showed that RBM4 overexpression or nPTB knockdown reduced the penetrating ability of HCT-8 cells through Matrigel and the membrane (Fig. 6B, upper). The number of RBM4-overexpressing or nPTB-KD HCT-8 cells invading through Matrigel was respectively reduced to ~55% or 29% of empty vector-transfected cells (Fig. 6B, lower). nPTB or miR-92a-2 overexpression consistently increased the invasive activity of HCT-116 cells to penetrate through the gel and membrane (Fig. 6B, upper); the number of invading HCT-116 cells respectively increased by ~4.1- or 4.8-fold with nPTB or miR-92a-2 overexpression compared to empty vector-transfected cells (Fig. 6B, lower). These results showed that the RBM4-regulated splicing cascade constituted a molecular mechanism which partially controlled the progression of CRC cells.

### RBM4 and nPTB exert opposite effects on the Warburg effect of CRC cells

**Figure 7:**
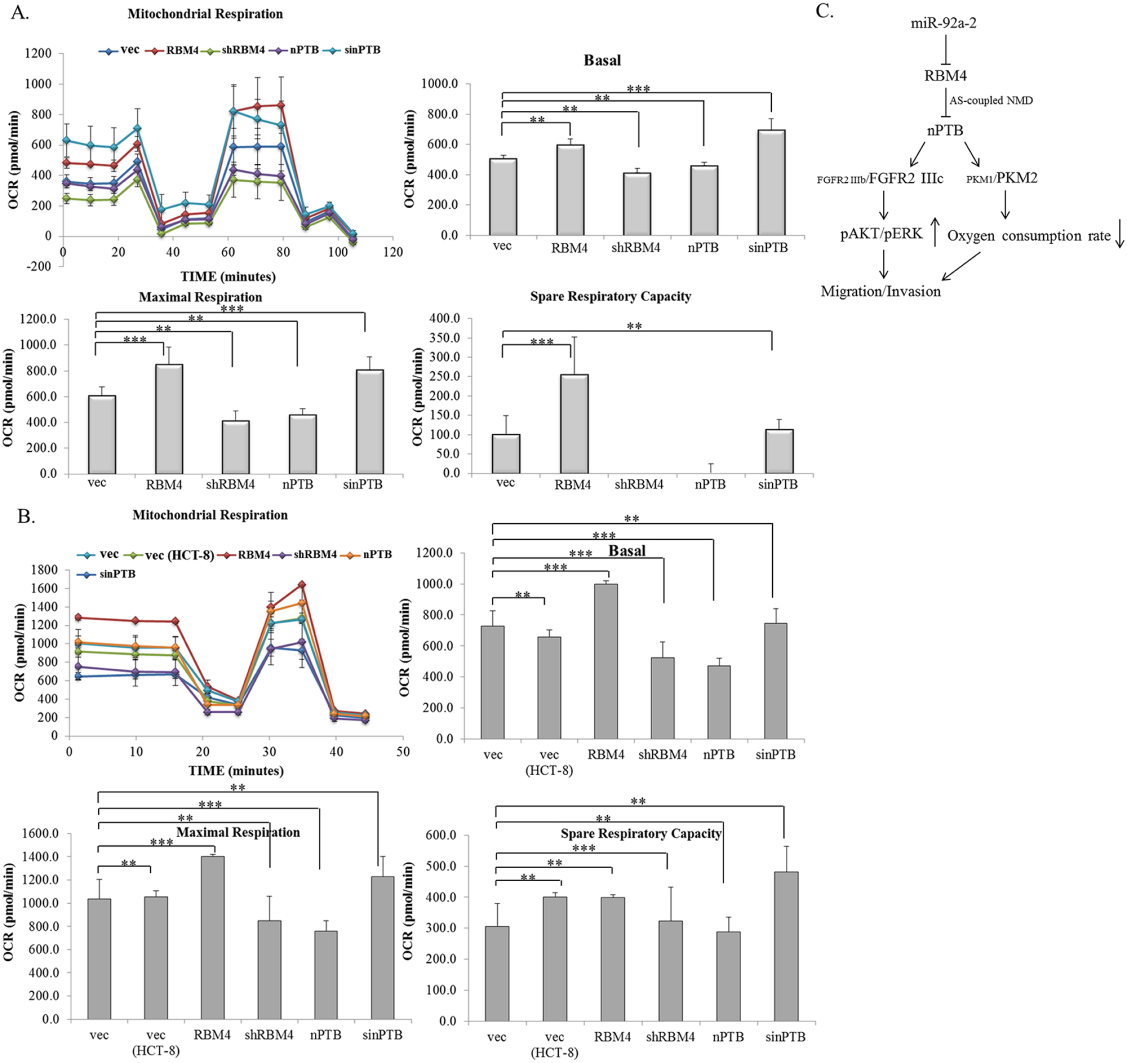
Expression profiles of RNA-binding motif 4 [RBM4] and neuronal polypyrimidine tract-binding protein [nPTB] modulate the mitochondrial activity of colorectal cancer [CRC] cells **A.** HCT-8 and **B.** HCT-116 cells were transfected with a parallel set of expressing or targeting vectors for 24 h. The basal and maximal oxygen consumption rates and spare respiratory capacity were measured using an XF20 Bioanalyzer [*n* = 4]. **C.** The RBM4-regulated splicing cascade was closely correlated with the cancerous signatures of CRC cells. Elevated miR92a-2 reduced the RBM4 level, which relieved its repressive effect on nPTB expression. The imbalanced expression of nPTB respectively enhanced relative levels of the *FGFR2 IIIc* and *PKM2* transcripts which modulated the progression and energy expenditure of CRC cells.

Compared to normal cells, cancerous cells exhibit active aerobic glycolysis, known as the Warburg effect [44, 45]. PKM2 was considered a key factor of this phenomenon which was closely correlated with the growth and progression of CRC cells [46, 47]. We next wondered whether the RBM4-regulated splicing cascade reduced the Warburg effect of CRC cells. As shown in Fig. 7A, overexpression of RBM4 or nPTB had a profound but opposite effect on the mitochondrial activity of HCT-8 cells by treating cells with complex-specific substrates. The analytic results showed that the basal oxygen consumption rate (OCR) was slightly elevated in RBM4-overexpressing or nPTB-knockdown HCT-8 cells compared to empty vector-transfected cells. On the contrary, the basal OCR of RBM4-knockdown or nPTB-overexpressing cells was slightly reduced (Fig. 7A, Basal). The maximal and spare capacities of the OCR were substantially upregulated in RBM4-overexpressing or nPTB-KD HCT-8 cells. Surprisingly, almost no spare respiratory capacity was left in RBM4-knockdown or nPTB-overexpressing HCT-8 cells (Fig. 7A, Maximal and Spare OCR). Results of parallel experiments were reproduced using HCT-116 cells. Elevated basal, maximal, and spare respiratory capacities were observed in RBM4-overexpressing or nPTB-knockdown HCT-116 cells, whereas RBM4-knockdown or nPTB-overexpressing cells exhibited reduced mitochondrial activity (Fig. 7B). The mitochondrial function was similar in distinct CRC cell lines. Taking the results together, RBM4 functioned as a tumor suppressor in reducing the progression and Warburg effect of CRC cells by reprogramming the CRC-associated splicing cascade.

## DISCUSSION

The expression levels of splicing factors directly control their biological activity on AS control [48]. The protein level and cellular localization of RBM4 was changed by multilayer mechanisms, including autoregulated AS, AS -coupled transcription control, and SRPK1-driven phosphorylation [17, 27, 28]. In this study, elevated miR-92a-3p constituted another mechanism for reducing RBM4 expression in CRC cells (Fig. 3C-F). These results indicated that RBM4 expression is meticulously controlled through complex mechanisms, since the influence of RBM4 could be expanded by initiating splicing cascades. RBM4 was previously demonstrated to reduce the protein levels of PTB and nPTB which constituted a cross-regulatory mechanism for the neuron development [20, 33]. Overexpressing PTB induced relative levels of the *PTB^−exon 11^* and *nPTB^−exon 10^* transcripts [33], but the nPTB-induced autoregulatory mechanism was first reported to enhance the expression of *nPTB^+exon 10^* transcripts (Fig. 3F). The feed-forward circuit could partially relieve the suppressive effect of elevated PTB protein on the expression of nPTB in CRC cells. Although mutual expressions of PTB and nPTB were observed during the development of neurons and skeletal muscles [33, 49], RBM4 was reported to repress expressions of PTB/nPTB via an AS-coupled NMD pathway in differentiating myocytes [14]. The regulatory effects of nPTB and PTB on the splicing profile were additive due to their cross-regulation, but RBM4 functioned as a dominant repressor in reducing the effects of nPTB/PBT proteins on AS events.

Imbalanced expression or localization of splicing factors leads to dysregulated splicing events, which are considered an occasion of cancerous progression [50, 51]. HnRNP A2/B1, PTB, and SRSF1 were reported to act oncogenes due to their influence of inducing cancer-associated transcripts [16, 52, 53]. In CRC cells, direct binding between elevated metastasis associated with lung adenocarcinoma transcript-1 (MALAT1) and the PTB-associated splicing factor (PSF) induced the dissociation of the nPTB/PSF complex, which promoted the progression of CRC cells. [35]. However, the molecular mechanism for the expression of nPTB protein in CRC cells was unknown. In this study, the miR-92a-caused reduction of RBM4 constituted a molecular mechanism that enhanced the expression of nPTB (Fig. 4C, lower). The regulatory effect of RBM4 on modulating nPTB/PTB expressions through an AS-coupled NMD pathway was previously reported in differentiating myocytes [14]. These results suggested that RBM4 functioned as a dominant repressor which reduced nPTB expression in a spatial-temporal manner, although more investigation is still required to verify this inference.

The FGFR2 and PKM isoforms were expressed spatiotemporally through AS mechanisms [54, 55]. The FGFR2 IIIb and PKM1 isoforms are exclusively expressed in differentiated cells, whereas FGFR2 IIIc and PKM2 are abundant in embryonic and cancerous cells [56, 57]. Increases in FGF2 and its receptor were widely observed in CRC tissues [58]. The relatively high expression FGFR2 IIIc led to the migration and invasion of cancerous cells [59]. In addition, HCT-8 cells exhibited an active EGF-EGFR response mediating the proliferation and progression of CRC, whereas HCT-116 was less responsive to EGF treatment [60]. Despite HCT-8 and HCT-116 being considered less-aggressive CRC cells and the invasive activity between these cells not having been experimentally evaluated, the relatively high level of *FGFR2 IIIc* transcripts suggested the active progression of HCT-8 compared to HCT-116 cells. Overexpressing RBM4 repressed the progression of various cancer cell lines [15], and our results showed that RBM4-modulated splicing cascade constituted a molecular mechanism regarding this phenomenon. In addition, the *FGFR2* gene was predicted as the specific candidate of RBM4 in a previous study [56]. Further investigation is required to verify this hypothesis in the future.

Dysregulated AS was considered a molecular hallmark of malignancy [61, 62]. Imbalanced expressions of FGFR2 IIIc and PKM2 isoforms contributed to the progression and metabolism of cancer cells [63, 64]. The mechanistic investigation of cancer-associated splicing events could bring new insights into the occurrence of distinct malignancies. In this report, we present a model of how RBM4 expression was reduced and its influence on the splicing cascade that participated in the cancerous signatures. Further investigation of the splicing factor and corresponding splicing events may provide a route for developing a useful biomarker and clinical therapy for various malignancies.

## MATERIALS AND METHODS

### Patient samples and cell culture

Human colorectal tumor samples (*n* = 20) were requested as anonymous specimens from the TMU Joint Biobank (approval no. 201409044). All of the recommendations of the Declaration of Helsinki for biomedical research involving human subjects were followed. The HCT-8, Colo205, and HCT-116 human colorectal adenocarcinoma and carcinoma cell lines were kind gifts from Dr. Yu-Chih, Liang (Taipei Medical University, Taipei, Taiwan). HCT-8, Colo205, and HCT-116 cells were cultured in RPMI-1640 medium supplemented with 10% fetal bovine serum, 600 mg/ml glutamine, 100 U/ml penicillin, and 100 mg/ml streptomycin (Invitrogen, Camarillo, CA, USA).

### Bioinformatic prediction of miRNA responsive element within *hRBM4* gene

To identify the putative miRNA target site within human *RBM4* gene, we used the publicly available algorithm, RNA22 V2.0 (https://cm.jefferson.edu/rna22/, 30) for the miRNA-*hRBM4* pair prediction. The upregulating profiles of CRC-related miRNAs were previously identified [29] and the convincing candidates (fold change >2, and *q* value <0.05; Supplementary Table 2) were included for the following prediction. The sequence of mature miRNAs was individually aligned with the sequence of *RBM4* 3′UTR or coding region in the RNA22 V2.0 algorithm. The parameter setting for RNA22 is: only 1 “UN-paired” base is allowed within the seed size of 7, minimum number of paired-up bases within predicted heteroduplex is 12, maximum value for the folding energy in any reported heteroduplex = −14 kcal/mol, and 2 wobble paired bases are allowed in seed region. The folding energy of putative miRNA-*hRBM4* pair was further predicted using RNAhybrid algorithm (http://bibiserv.techfak.uni-bielefeld.de/rnahybrid). A lower *p* value generated by these programs represented a higher possibility that the aligned target sequence contained a valid miRNA responsive element. The miRNA target pairs with low folding energy (<-14 kcal/mol) and convincing *p* value (<0.005) were subjected to the further investigation.

### Plasmid construction and transfection

The pRL-hRBM4 open reading frame (ORF) reporter was constructed by inserting the human RBM4 ORF fragment (301~652 nt) into the pRL-SV40 vector (Promega, Madison, WI, USA). The human RBM4 ORF was polymerase chain reaction (PCR)-amplified using a complementary (c)DNA library prepared from fetal brain tissue as the template and then inserted into *Xba* I/*Not* I sites of the pRL-SV40 vector. To express human miR-92a-2 RNA, the PCR product coding for its primary sequence was cloned into the pSilencer 1.0-U6 vector (Invitrogen). The *nPTB* minigene reporter was constructed by inserting the complete human *nPTB* genomic fragment containing exons 9, 10, and 11 and intra-exon introns. The genomic fragments were amplified by a PCR using genomic DNA prepared from MRC5 fibroblasts as the template and then inserted into *Kpn*I/*EcoR*V sites to replace the β-galactosidase gene of pCH110 (Amersham Pharmacia, Uppsala, Sweden). Mutant pRL-hRBM4 ORF, pCH-nPTB, and pSilencer-miR-92a-2 vectors containing changed nucleotides were constructed using the QuikChange site-directed mutagenesis system (Stratagene, Amsterdam, Netherlands). Sequences of the PCR primer sets are listed in Supplementary Table 3. All constructs were auto-sequenced. Cultured cells were grown to 60% confluence, and the indicated plasmid was transfected using PolyJet (SignaGen Laboratories, Ijamsville, MD, USA). After 24 h, total RNA and proteins were separately extracted using the Trizol reagent (Invitrogen). For the reverse-transcription (RT)-PCR assay, 2 μg of RNA was reverse-transcribed using SuperScriptase III (Invitrogen) in a 10-μl reaction. The PCR analysis of individual genes was performed using gene-specific primer sets (Supplementary Table 2). The PCR-amplified amplicons of *PKM* and *FGFR2* were then digested with *Pst*I and *Eco*RV to discriminate the products containing *PKM2* exon 10 and *FGFR2IIIc* exons. Densities of the PCR products were determined using TotalLab Quant Software. A quantitative (q)RT-PCR was performed with SYBR green fluorescent dye and gene-specific primer sets using an ABI One Step™ PCR machine (Applied Biosystems, Foster City, CA, USA). The relative messenger (m)RNA level was quantitated by the ΔΔ-Ct method, and the level of GAPDH mRNA served as the internal control.

### Immunoblot assay

The immunoblot analysis was conducted using an enhanced chemiluminescence (ECL) system (Millipore, Billerica, MA, USA), and images were analyzed with the LAS-4000 imaging system (Fujifilm, Tokyo, Japan). Primary antibodies used in this study included polyclonal anti-RBM4 (Santa Cruz Biotechnology, Santa Cruz, CA, USA), monoclonal anti-nPTB (Abnova, Taipei, Taiwan), polyclonal anti-GAPDH (MDBio, Taipei, Taiwan), monoclonal anti-FLAG M2 (Sigma-Aldrich, St. Louis, MO, USA), polyclonal anti-ERK1/2, polyclonal anti-AKT, monoclonal anti-phospho-ERK1/2 and monoclonal anti-phospho-AKT (Cell Signaling Technology, Danvers, MA, USA).

### RNA electrophoretic mobility shift assay (REMSA)

Recombinant His-tagged proteins were prepared as described previously.^17^ RNA probes used were *nPTB* exon 10 (CU, 34 nt) and the downstream intron (86 nt). Exonic and intronic elements were *in vitro*-transcribed and used as probes. For RNA-protein interactions, 1 or 4 μg of recombinant protein was incubated with 10 nM of DIG-labeled probe in a 20-μl reaction containing 10 mM HEPES (pH 7.9), 50 μM EDTA, 10% glycerol, 1 mM dithiothreitol, 5 mM MgCl_2_, 0.5 μg/ml bovine serum albumin, and 12.5 ng/ml tRNA for 15 min at room temperature. Reactions were analyzed by electrophoresis on an 8% nondenaturing polyacrylamide gel in TBE buffer (45 mM Tris-HCl, 45 mM boric acid, and 1 mM EDTA; pH 8.0). Binding complexes were transferred to nylon membranes (Hybond N, Amersham Bioscience, Piscataway, NJ, USA) that were irradiated under 254-nm light for 60 s. Immunoblotting was conducted by incubating membranes with horseradish peroxidase (HRP)-conjugated anti-DIG Fab fragments (Roche, Mannheim, Germany).

### Poly(A) tailing of small RNA

Small RNAs were poly(A)-tailed by A-Plus Poly(A) polymerase (Cellscript, Madison, WI, USA) as per the user's instruction manual. In brief, 50 μg of total RNA was preheated to 65°C for 10 min. The RNAs were then incubated with 8 U A-Plus Poly(A) polymerase in a 100-μl mixture at 37°C for 60 min. The reaction was immediately stopped by storing the mixture in a −70°C freezer. Tailed-RNAs were extracted using a PCA/ethanol method. The RNA pellet was dissolved in nuclease-free water and subjected to an RT-PCR assay using specific primer sets (Supplementary Table 3).

### *In vivo* translation assay

HCT-8 cells were seeded in six-well plates (2 × 10^5^ cells/well) 24 h prior to transfection. The transfection reaction mixture contained 0.5 μg of the pRL-SV40 and the engineered *Renilla* luciferase reporters which contained a wild-type (WT) or mutant h*RBM4* ORF fragment, 1 μg of the effector expression vector, and 0.5 μg of the pFir-Luc vector (Promega) as the control. After 24 h, transfectants were lysed using passive lysis buffer, and cell debris was removed after centrifugation. Activities of the firefly and Renilla luciferases were measured using a dual-luciferase assay kit (Promega) and the Synergy HT multi-mode microplate reader (BioTek, Winooski, VT, USA).

### *In vitro* translation assay

pRL-hRBM4 and pGL3-Basic vectors were linearized and used as templates for *in vitro* transcription; these transcripts were diguanosine-capped and had a short poly(A) tail. The transcribed RNA was precipitated using LiCl (7M) and quantified by Nanodrop spectrophotometry. The cytoplasmic fraction (S100) was extracted from miR-92a-2-3p- or miR-92a-2m3p-overexpressing HCT-116 cells as previously described (26). The abundance of total miR-92a was estimated using a qRT-PCR. The *in vitro* translation reaction (25 μl) contained 5 ng of *in vitro*-transcribed RNA, 150 pM of miR-92a, 25 μM amino acids, and 12.5 μl reticulocyte lysate (Promega). The mixture was incubated at 30°C for 2 h, followed by subjecting 10% of the mixture to the luciferase assay as described above. Each reaction was independently performed at least three times.

### Mitochondrial respiration assay

A Seahorse XF24 extracellular flux analyzer (Seahorse Bioscience, North Billerica, MA, USA) was used to measure the oxygen consumption rate (OCR; as an indicator of mitochondrial respiration). In brief, 5 × 10^4^ HCT-8 cells were seeded in each well of Seahorse XF24 plates with 250 μl of Dulbecco's modified Eagle medium (DMEM) and incubated overnight. Prior to the measurement, cells were washed with unbuffered medium and immersed in 675 μl of unbuffered medium without CO_2_ for 1 h. The OCR was assessed in 8-min cycles as recommended by Seahorse Bioscience. The basal and maximal OCRs, and spare respiratory capacity were recorded following injection of complex-specific substrates, including FCCP (0.5 μM), rotenone (2 μM), and oligomycin (2.5 μg/ml).

### *In vitro* migration and invasion assay

HCT-8 and HCT-116 cells (5 × 10^4^ cells/well) transfected with distinct effector expression vectors were seeded in the top chamber containing a polycarbonate membrane (8-μM pore, Corning, Cambridge, MA, USA). The lower chamber contained complete culture medium which served as a chemoattractant. After 24 h, the inner membrane was scraped with a swab, and cells which had migrated to the lower side of the membrane were fixed with 4% paraformaldehyde and stained with Giemsa solution for counting. For the *in vitro* invasion assay, transwell chambers were coated with Matrigel prior to cell seeding. After 24 h, the inner membrane was scraped with a swab, and cells which had invaded were fixed with 4% paraformaldehyde and stained with Giemsa solution for counting. Numbers of migrating and invading cells were counted in four high-power fields (HPFs).

## SUPPLEMENTARY TABLES AND FIGURE


