# Supplementary material for: The impact of RNA binding motif protein 4-regulated splicing cascade on the progression and metabolism of colorectal cancer cells
Source: Oncotarget. 2015 Oct 19;6(35):38046–60. doi: 10.18632/oncotarget.5710 (PMC4741983; doi:10.18632/oncotarget.5710)
Supplement: Supplementary file 1 [file oncotarget-06-38046-s001.pdf]

SUPPLEMENTARY TABLES AND FIGURE

Supplementary Table S1: Putative targeting miRNAs of RBM4

<table>
<thead>
<tr><th>miRNA</th><th>Alignment</th><th>Leftmost position of predicted site</th><th>Folding energy (Kcal/mol)</th><th>p Value</th></tr>
</thead>
<tbody>
<tr><td>has-miR-103a</td><td>CAGGAGCTGCTGCCACAGCTGCTGCT
|||: ||| |||: |||||||
AGTATCGG-GAC-ATGTT-ACGACGA</td><td>+837</td><td>–14.0</td><td>5.61E-3</td></tr>
<tr><td>hsa-miR-18a</td><td>CCAGAATACAGC-C-ATGGCCAGT
|||||| ||| | || ||||
GGTCTT-CCTCGTGAATCCCGTCA</td><td>+763</td><td>–15.2</td><td>2.56E-2</td></tr>
<tr><td>hsa-miR-92a</td><td>ACATGGAGCGGGCAGAGGATGCAGTG
||| || |||| | |:| ||||:|:
TGT-CC-GGCCC-TGTTC-ACGTTAT</td><td>+348</td><td>–19.2</td><td>5.23E-2</td></tr>
<tr><td>hsa-miR-23a</td><td>GGAAGGTGCTGG-AATGTGAC
||||: : |||| |||||||
CCTTTAGGGACCGTTACACTA</td><td>+75</td><td>–18.8</td><td>3.21E-1</td></tr>
<tr><td>hsa-miR-139</td><td>GCGCTA-CAGCCCC-AGTCCCCA
:| |:| ||| || ||| |||
TGAGGTTGTCCCGGCGCAGAGGT</td><td>+936</td><td>–14.6</td><td>2.3E-2</td></tr>
<tr><td>hsa-miR-222</td><td>TAGA-AGACAAGACGGCAGCTGAG
|| ||| ||| :|||||
TCCTAGATGT--GACCGATGACTC</td><td>+123</td><td>–14.2</td><td>9.43E-2</td></tr>
<tr><td>hsa-miR-324-5p</td><td>ACAAGACGGCAGCTG-AGGATGCC
||| || ||: ||||||
TGTGGTTACG-GGATCCCCTACGC</td><td>+129</td><td>–14.2</td><td>9.43E-2</td></tr>
<tr><td>hsa-miR-214</td><td>GATGCCTACTACAAGCGCTGCCGT
||||| || |||| ||
TGACGGA-CAGACACG-GACGACA</td><td>+653</td><td>–14.2</td><td>3.47E-1</td></tr>
<tr><td>hsa-miR-25</td><td>ATGGAGCGGGCAGAGGATGCAGTG
:|| ||:| | |:| ||||:||
AGTCTGGCTC-TGTTC-ACGTTAC</td><td>+350</td><td>–18.7</td><td>5.23E-2</td></tr>
<tr><td>hsa-miR-24</td><td>TCCCCACTGTTGGAGAGGGCTA
|||:||:| |:||:|
GACAAGGACGACTTGACTCGGT</td><td>+951</td><td>–16.4</td><td>2.3E-2</td></tr>
<tr><td>hsa-miR-17</td><td>CCGCTGCTGCTGTTACTGCAGC
| ||: |:||||||
GATGTTCACGGAAGTGACGTCA</td><td>+873</td><td>–17.6</td><td>2.92E-2</td></tr>
<tr><td>hsa-miR-17</td><td>CTGCCTCCTGCCT-C-CTGCGGC
||:| ||||| | ||||:|
GATG-TTCACGGAAGTGACGTCA</td><td>+1346 (3‘UTR)</td><td>–24.1</td><td>4.85E-3</td></tr>
</tbody>
</table>

Supplementary Table S2: List of upregulated miRNAs in CRC tissues

Candidates	Reference
miR-92a<br>miR-21<br>miR-29a<br>miR-125<br>miR-196<br>*miR-103*<br>miR-135b<br>miR-26b<br>miR-182	Int J Colorectal Dis, 28, 19–24<br>Eur J Cancer, 51, 409–420<br>Cancer Biomark, 15, 103–113<br>Eur J Cancer, 51, 409–420<br>World J Gastroenterol, 18, 2827–2831.<br>Int J Mol Sci. 2014 May 13;15(5):8458–8472<br>Am J Cancer Res, 5, 1382–1395<br>J Cell Mol Med, 15, 1941–1954<br>Oncol Rep, 33, 2592–2598
miR-206<br>1miR-7	Oncol Rep, 33, 1402–1410<br>Carcinogenesis, 36, 338–345
miR-320b	Eur J Cancer, 51, 409–420
miR-193a	World J Gastroenterol, 20, 12241–12248
miR-708	Oncol Lett, 8, 1768–1774.
miR-31	Oncol Lett, 8, 1768–1774.
miR-18a	Cancer Biomark, 15, 103–113
miR-155	Tumour Biol, 36, 1619–1625
miR-181a<br>amiR-106a<br>miR-15a-3p<br>miR-19<br>*miR-23a*<br>miR-224<br>miR-200b<br>miR-200c<br>miR-124<br>miR-30<br>*miR-214*<br>miR-455-3p<br>*miR-17*<br>miR-128<br>1miR-130b<br>bmiR-152	J Clin Pathol, 67, 198–203<br>FEBS Lett, 588, 705–712.<br>PLoS One, 10, e0132495<br>Mol Cancer Res, pii: molcanres.0466.2014.<br>Int J Mol Sci, 15, 11713–11729.<br>Br J Cancer, 112, 1480–1490<br>RNA Biol, 12, 276–289<br>RNA Biol, 12, 276–289<br>Biochim Biophys Acta,. pii: S0925–4439<br>Mol Cell Biol, 35, 988–1000<br>Hum Mutat, 33, 551–560<br>PLoS One, 9, e109430<br>Oncotarget, 5, 2974–2987<br>Oncotarget, 6, 5412–5425<br>Neoplasia, 15, 1218–1231<br>Mol Cell Biochem, 345, 283–290
miR-34b<br>*miR-25*<br>miR-20b<br>*miR-24*<br>miR-18b	Med Oncol, 31, 894<br>Int J Mol Med, 34, 1286–1292<br>Hum Immunol, 75, 348–353<br>Eur J Cancer, 51, 409–420<br>Cancer Res, 70, 8233–8246
miR-93-5p	PLoS One, 8, e83025
miR-324-5p	Unknown
miR-139-3p	Oncol Rep, 25, 739–747
miR-222-5p	Exp Cell Res, 318, 2168–2177

Supplementary Table S3: List of PCR Primers

Gene	Forward	Reverse	cycle
nPTB	gcatttgccaaggagacatcc	cgctgcacatctccataaacac	35
nPTB(minigene)	atcggtaccggcttcctgttgcagctgttc	atcgatatccaagttgtgattggtttcc	30
nPTB(minigene mut)	aaagtctgtttaccggcttcggtatgttattg	caataacataccgaagccggtaaacagacttt	18
hRBM4 (ORF)	atctctagatggtccggtcatcgaatgtg	atcgcggccgcgcgctccgtacgcgttgttg	30
hRBM4 (ORF mut)	cgggcagaggatgcaggggaggccatcaggggc	gcccctgatggcctcccctgcatcctctgcccg	18
miR-92a-2	tattgcacttgtcccggcctgt		
miR-92a-2 (WT)	gtgaattcctgtgtgatattctgctg	ggctcgagtgccaaatctgacacgc	35
miR-92a-2 (m5P)	accccttctacacatgttgggatcggttgc	gcaaccgatcccaacatgtgtagaaaggggt	10
miR-92a-2 (m3P)	gtgtttctgtatggtcttgcacttgtcccgg	ccgggacaagtgcaagaccatacagaaacac	18
SV40miR-polyA(R)	ttttggaggcctaggctttt	ccgaggcggccgacatgttt	35
FGFR2	ggatcaagcacgtggaaaagaac	ggcgattaagaagacccctatgc	35
PKM1/2	agaaacagccaaaggggact	cattcatggcaaagttcacc	35
GAPDH	cggagtcaacggatttggtcgtatg	agccttctccatggtggtgaagac	30
RBM4	taggatccgcagtgcgtac	ttaaaaggctgagtccgcg	35
16S rRNA	agagtttgatcmtggctcag	ggmtaccttgttacgactt	
Pri-miR-92a-1	ctttctacacaggttg	caaactcaacaggc	
Pri-miR-92a-2	tctccagcactcatgcc	ctttcttccacaggccg	

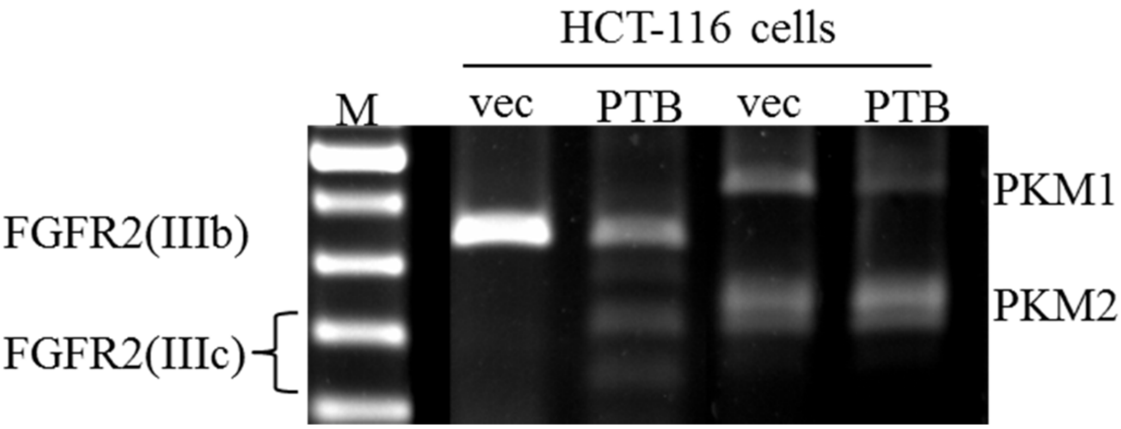

Supplementary Figure S1: Polypyrimidine tract-binding protein (PTB) modulated the splicing patterns of the *FGFR2* and *PKM* genes HCT-116 cells were transfected with empty vector or PTB expressing vector, followed by total RNA extraction. Splicing profiles of FGFR2 and PKM pre-mRNAs were analyzed as described in “Materials and methods” section.
